# Students’ perceptions of endodontic typodont teeth with simulated canals printed from novel materials

**DOI:** 10.3389/fdmed.2024.1373922

**Published:** 2024-06-14

**Authors:** Alexander Jon Cresswell-Boyes, Graham Roy Davis, Aylin Baysan

**Affiliations:** ^1^ Peninsula Dental School, Faculty of Health, University of Plymouth, Plymouth, United Kingdom; ^2^ Dental Physical Sciences Unit, Centre for Oral Bioengineering, Institute of Dentistry, Barts and the London School of Medicine and Dentistry, Queen Mary University of London, London, United Kingdom; ^3^ Centre for Oral Bioengineering, Institute of Dentistry, Barts and the London School of Medicine and Dentistry, Queen Mary University of London, London, United Kingdom

**Keywords:** dental education, 3D printing, endodontics, simulation-based education, haptic (tactile) perception

## Abstract

**Introduction:**

This study aimed to investigate students' perceptions of the use of 3D-printed typodonts by implementing a questionnaire and evaluating the students' comparisons between extracted, commercial and 3D-printed teeth.

**Methods:**

Ethical approval was obtained (QMER20.586/2021) and questionnaire feedback was collected anonymously using an online survey. A total of 143 fourth- and fifth-year dental students were approached to participate during pre-clinical courses focussing on root canal therapy. The tooth design was based on micro-CT data of an extracted maxillary central incisor and 3D-printed with haptically-similar materials produced in previous work. The questionnaire comprised 11 Likert-scale questions, four open-ended questions, two “yes” or “no” questions and three closed-ended questions.

**Results:**

Eighty questionnaires were returned. Overall, the feedback was favourable towards the 3D-printed typodonts compared to the commercial teeth. The biggest difference in responses was in Question 6 related to the realism of drilling the enamel when comparing 3D-printed teeth with commercial ones. Statistical analysis showed a significant difference (*p* < 0.05); the fourth-year's response on average, was 2.95 (±0.73) an “agree” rating, whereas the fifth-year's response was 3.98 (±0.82) with “neither agree or disagree”.

**Discussion:**

Within the limitations of this study, the 3D-printed typodonts were rated high in comparison to the commercial teeth in terms of overall operative experiences.

## Introduction

Clinical endodontics requires dentists to become competent in a wide range of manual tasks such as access cavity preparation, identifying the canal orifices as well and shaping and obturating the root canal systems (1–3). Undergraduate dental students must carry out these repetitive practical tasks typically on extracted teeth before seeing patients (4) since root canal therapy using extracted teeth would provide a realistic training scenario (5). However, there are well-documented drawbacks i.e., limited availability, cross-infection risks, ethical approval and no standardisation (6–8). Therefore, typodonts have been used as an alternative, as well as in conjunction with, extracted teeth. The simulated root canal systems could overcome the drawbacks of extracted human teeth, as they are realistic and standardised with unlimited availability (9–11). However, the use of typodonts for training purposes is seen as a debatable aspect of endodontic education from the didactic point of view.

In this respect, Tchorz et al. (7) stated that such typodonts would prepare dental students for clinical settings as effectively as extracted human teeth. However, Bitter et al. (8) reported that training on tooth replicas might not accurately predict student performances when the dental students treated patients in the clinic. In particular, these authors demonstrated that the properties of typodonts were different in comparison to the extracted teeth. Their mechanical properties, as well as their “feel”, have been rated as not being comparable with that of real human teeth (6, 12). Cresswell-Boyes et al. (13) established a method of quantifying the “feel” of cutting natural tissue compared to commercial typodonts by measuring the force exhibited from a high-speed dental handpiece. The authors concluded that commercial tooth replicas were not comparable to natural dental hard tissues, offering an unrealistic haptic experience. Interestingly, some tooth replicas required more than double the force to prepare, in one instance the artificial dentine required a force of 1.85 N (One Dental, Australia) to cut, with natural dentine requiring an average of 0.49 N. It was concluded that composite resins used with 3D printing might be an alternative material of choice, despite not having the same mechanical properties, however, the feel was similar to the extracted teeth (13). Reymus et al. (14) also concluded similar findings, that despite the lack of similar hardness to the natural teeth, there were no differences detected during mechanical instrumentation of 3D-printed root canal systems which were made from commercial resins.

Therefore, the current 3D-printing technology could be promising to enhance student learning in dental education, especially for clinical endodontics (15–17). Recently, Höhne et al. (18) designed teeth with realistic carious lesions and dental pulp cavities using 3D printing to create different enamel (Rigid Resin, Formlabs Inc., United States) and dentine (White Resin, Formlabs Inc., United States) layers. Hanafi, Donnermeyer et al. (19) produced a modular 3D-printed training model (Dental Model Resin, Formlabs Inc., United States) allowing for both extracted teeth and typodonts to be embedded. Additionally, macro-models were produced utilising transparent resin (Formlabs Inc., United States) in Pouhaër et al. (3) for undergraduate preclinical practice for endodontic access cavities. All three studies utilised a stereolithography 3D printer, Formlabs Form 2 (Formlabs Inc., United States).

Using multi-jet modelling (Stratasys Objet30 Dental Prime, Stratasys Ltd., United States), Tonini, Xhajanka et al. (20) produced CBCT-generated models using transparent materials (VeroClear, Stratasys Ltd., United States). Similarly using the same printer, Kolling et al. (21) 3D-printed typodont models (VeroWhitePlus, Stratasys Ltd., United States), that imitated a Vertucci Class V root-canal (22), in which the main canals are divided in the middle third, into two separate canals. The authors used the models within an evaluation study with 88 students, in which students’ perceptions and educational environment were assessed. Overall, the 3D-printed teeth received significantly lower ratings when compared to human teeth, regarding, enthusiasm, the learning of fine motor skills and spatial awareness. Despite this, however, students noted as having the benefits of cleanliness, availability, and the standardisation of training on complex root-canal configurations. The authors reported that the physical characteristics of the 3D-printed teeth are what prevented them from rating them higher than extracted teeth, and suggested that improvements in this respect, along with its educational advantages would enhance learning opportunities (21).

Despite these innovative new teaching tools, the objective of dental education is to produce competent clinicians whilst also promoting student enthusiasm for using these tools. Feedback for the use of these new educational approaches is crucial in understanding what demands need to be met in production. Therefore, this work aims to offer an alternative to materials previously published, that are haptically similar to natural teeth. This study aimed to investigate the difference between fourth- and fifth-year students' perceptions of the use of haptically-similar 3D-printed typodonts as produced in Cresswell-Boyes et al. (13) and assess the produced typodonts' viability as an endodontic teaching tool by implementing a questionnaire and evaluating the students' comparisons between extracted, commercial and 3D-printed teeth.

## Materials & methods

### Ethical approval and data protection

Ethical approval for the study was obtained from the Queen Mary Ethics of Research Committee (QMER20.586/2021), with the feedback being collected anonymously using the Online Surveys (formerly BOS; JISC, United Kingdom) platform. Students consented before participation and were informed that their participation was not linked to their academic progress. Data were processed and stored following current data protection laws.

### Questionnaire development

The questionnaire was developed and adapted following the results of a pilot study involving clinically qualified dental educators.

There were 11 Likert-scale questions with four open-ended, two yes or no and three closed-ended questions (Table 1). The questionnaire aimed to compare the 3D-printed typodonts with extracted and commercial plastic teeth (Real T Endo, Acadental, Inc., USA), predominantly used in the Endodontic course at Queen Mary University of London. At the end of the questionnaire, students were also able to enter free-text comments.

**Table 1:** Questionnaire developed for the study.

No.	Question
1.	Starting time
2.	Finishing time
3.	The anatomical details of the 3D-printed endodontic tooth with a simulated canal are similar to an extracted tooth
4.	The anatomical details of the 3D-printed endodontic tooth with a simulated canal are similar to commercially available Endo plastic tooth with a simulated canal
5.	The likeness of drilling the enamel (feel) is similar to an extracted tooth
6.	The likeness of drilling the enamel (feel) is similar to an Endo plastic tooth with a simulated canal
7.	The likeness of drilling the dentine (feel) is similar to an extracted tooth
8.	The likeness of drilling the dentine (feel) is similar to an Endo plastic tooth with a simulated canal
9.	The likeness of exposing the dental pulp (feel) for root canal treatment is similar to an extracted tooth
10.	The likeness of exposing the dental pulp (feel) for root canal treatment is similar to an Endo plastic tooth with a simulated canal
11.	The 3D-printed tooth with a simulated root canal's overall value in Endodontic experiences and training is similar to an extracted tooth
12.	The 3D-printed tooth with a simulated root canal's overall value in Endodontic experience and training is similar to Endo plastic tooth with a simulated canal
13.	The 3D-printed tooth with a simulated root canal is less prone to errors i.e., perforation, over preparation of root canal system in comparison to Endo plastic tooth with a simulated canal
14.	How long did it take you to do the access cavity?
15.	Would you use this tooth in endodontics CSL technique courses?
15a.	If no, please state the reasons
16.	Would you prefer having this tooth in assessments (including gateway and revalidation tests)?
16a.	If no, please state the reasons
17.	What aspect of the model was your most favourite?
18.	What aspect of the model was your least favourite?
19.	Please feel free to add any other comments

Questions 3–13 were 5-point Likert-scale questions ranging from “strongly agree”, “agree”, “neither agree or disagree”, “disagree”, and “strongly disagree”.

The Likert-scale questions were scored on a 5-point scale, with numerical values added afterwards for data analysis: “strongly agree” (1), “agree” (2), “neither agree or disagree” (3), “disagree” (4) and “strongly disagree” (5).

### Fabrication of the 3D-printed teeth

**Figure 1:**
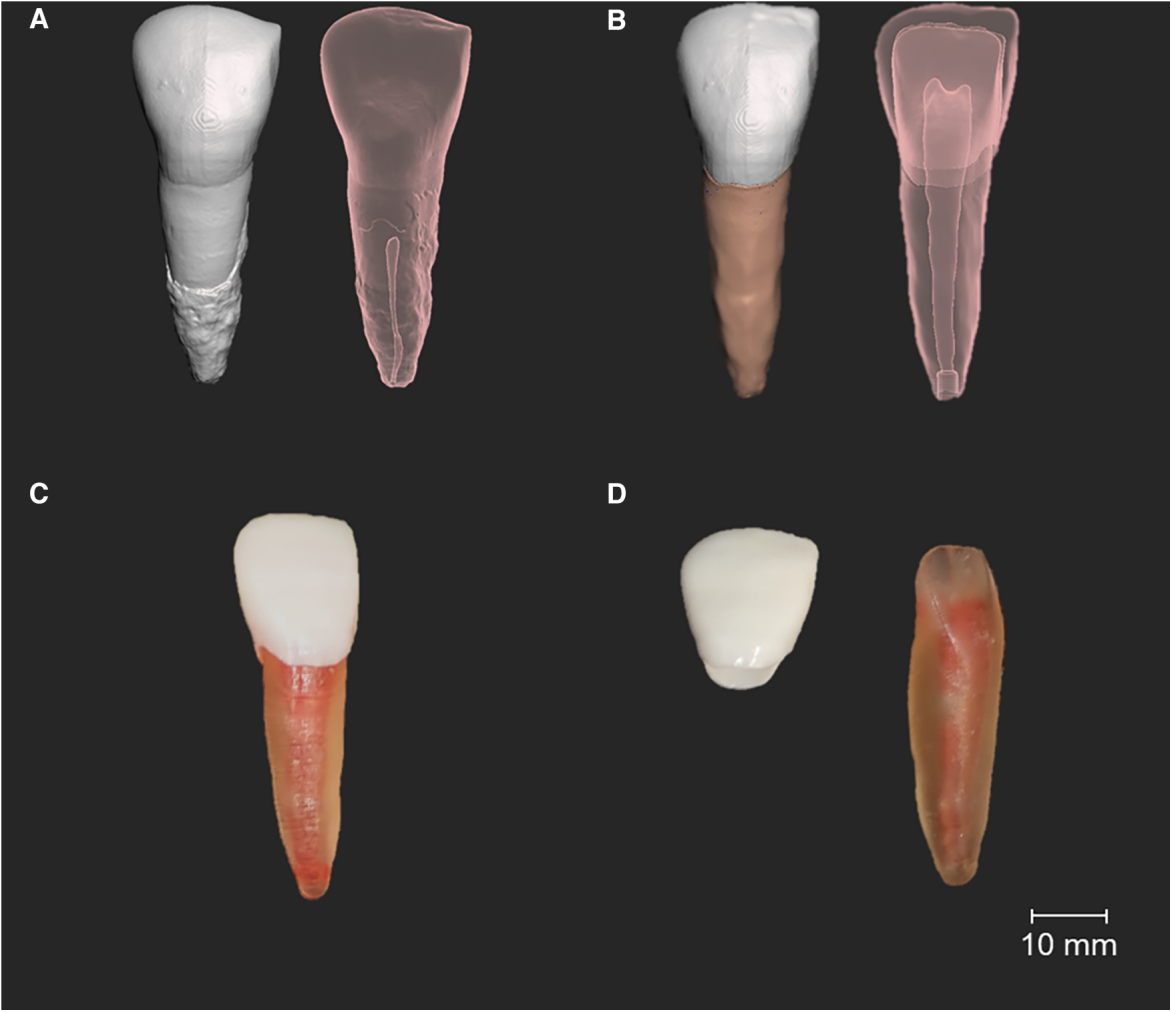
Manufactured 3D-printed typodont with a simulated root canal system. (**A**) Initial micro-CT generated model, opaque and transparent view, showing the reabsorbed root canal system. (**B**) A segmented model with the redesigned root canal system, showing additional access canals added to insert the synthetic system. (**C**) 3D-printed typodont fixed together. (**D**) Different 3D-printed structures before fixation.

**Figure 2:**
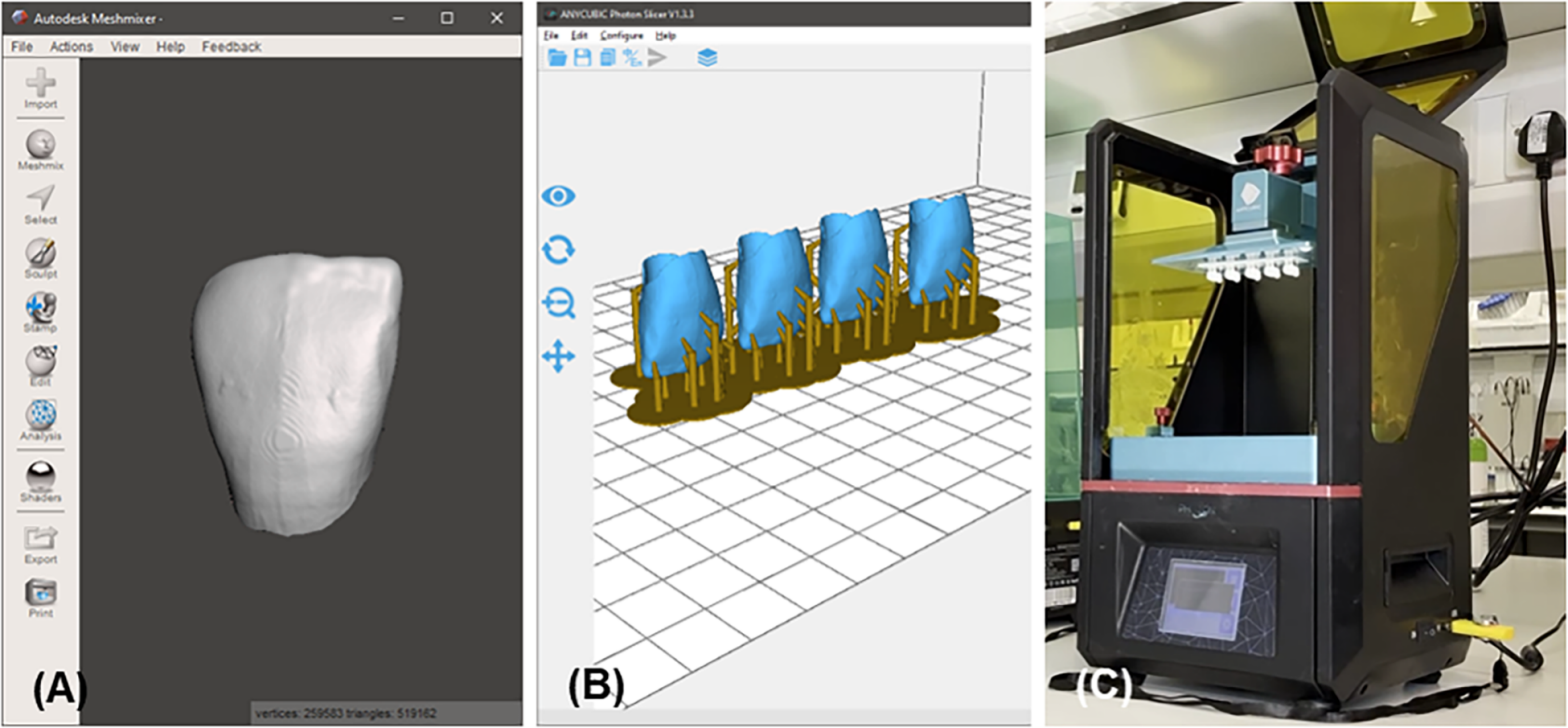
The 3D printing process (**A**) meshmixer view of the segmented enamel. (**B**) Anycubic Photon Slicer view of the segmented enamel. (**C**) 3D printing of the segmented enamel.

The tooth design was based on micro-CT data of an extracted maxillary central incisor (23). The extracted tooth chosen had the desired external anatomy, however, the pulp cavity had been reabsorbed (Figure 1A). The root canal system was created using Meshmixer [Version 2.2, 2016; Autodesk Inc., USA (Figure 2A)], based on the dental pulp anatomy, and the root canal system was classified as Type I according to Vertucci's root canal system configuration (22, 24).

Due to enamel wear on the extracted incisor tooth (Figure 1A), the enamel part was extended and straightened off by providing a sharp incisal edge using Meshmixer, according to the anatomy of central incisor teeth (Figure 1B).

Subsequently, an access canal was added to the apex of the root and palatal surface (Figure 1B) to allow the injection of ribbon wax (Metrodent, UK) to mimic the root canal system. The process of segmentation of the differing structures is outlined in Cresswell-Boyes et al. (23).

An Anycubic Photon 3D printer [Anycubic, China (Figure 2C)] and it's proprietary slicing software [Anycubic Photon Slicer, Version 1.3.3, 2018, Anycubic, China (Figure 2B)] was then utilised in the production of the 3D-printed typodonts with the dentine and enamel composed of carbonated hydroxyapatite resins (5 wt.% and 20 wt.% respectively) with Anycubic White Resin (Anycubic, China). The fabrication of the resins and 3D-printed typodonts are outlined in Cresswell-Boyes et al. (13). Carbonated hydroxyapatite was produced as outlined in Landi et al. (25), the subsequent powder was milled using a Gy-Ro mill (Glen Creston, UK) for 10 min, before being sieved through a <38 μm stainless steel sieve (Endocotts Ltd., UK). The carbonated hydroxyapatite was then added to the Anycubic White Resin at different weight percentages (5 and 20), the mixture was mechanically mixed for 24 h at 37°C to allow for complete dispersion. The mixture was placed within an opaque container to ensure no curing took place before printing.

Some of the 3D-printed typodonts lacked the dental pulp cavity, due to the limitations of direct light processing technology. As the dentine was manufactured using a translucent resin, each tooth produced in this study was visually checked for the presence of a cavity. Once printed, the dentine was washed through with 90% ethanol (via the access canal), to remove any uncured resin in the pupal cavity. Ribbon wax was heated to 50.0°C and injected with the wax through the access canals into the pulpal cavity to provide the root canal systems and subsequently filled with uncured “dentine” resin. The enamel layer was then fixed to the dentine with uncured “enamel” resin and cured for a period of 10 s using a handheld curing light (Elipar^TM^ DeepCure-S, 3M, USA) (Figure 1C).

### Data collection

The study took place with fourth- and fifth-year dental students undertaking the Advanced component of the Endodontics Speciality course using typodonts in phantom heads. Fourth- and fifth-year students were selected due to their enrolment in the speciality course, as well as their experience in performing procedures on extracted and commercial typodont teeth. Fourth-year students at this point would have 2 years of experience, whereas fifth-year students would have 3 years. During the pre-clinical courses, undergraduate dental students performed root canal treatments on central incisors with simulated root canal systems (Real T Endo, Acadental, Inc., USA) and the 3D-printed typodont teeth using rotary files (ProTaper Gold, Dentsply Sirona, USA). Wax blocks were given with both sets of teeth, allowing the mounting of the teeth. Participants were encouraged to perform either trepanation or a complete endodontic procedure, based on their discretion.

The outline of this study was explained to the students by providing participant information sheets (PIS). Students were then approached and requested to sign the consent forms if they agreed to participate. These participants were requested to evaluate their training experiences with the 3D-printed typodonts. The PIS and consent forms were then kept in the locked cupboard.

### Quantitative and qualitative analysis

Statistical analysis was conducted using Microsoft Excel (Version 1909, 2019; Microsoft, USA). An analysis is presented as mean ($\bar{x}$), minimum (Min) and maximum (Max) and standard deviation (SD). Statistical differences were analysed using Welch's *t*-test. Free-text comments were analysed thematically.

## Results

### Students' feedback and perceptions

Out of 143 students, 80 questionnaires were returned (37 fourth-year, and 43 fifth-year students), giving a 55.94% participation rate. Students, on average, spent around 5–15 min with the 3D-printed typodonts provided, this was down to the participants' discretion of how they performed the root canal treatment. Some participants only generated an access cavity to the pulpal chamber, whereas others performed a complete root canal treatment.

Table 2 shows the results from the questionnaire. Overall, the results of the questionnaire were typically favourable towards the 3D-printed typodonts compared to the commercial teeth that participants were more familiar with. Question 3 regarding the anatomical details of the 3D-printed typodont compared to extracted teeth, overall, had the highest response for “strongly agree” (1) with an overall score of 1.46 (±0.16). Whereas, Question 10, elicited the strongest response for “disagree” (4) with an overall score of 4.09 (±0.49), in response to the similarity between the 3D-printed tooth to the commercial tooth in regards to exposing the pulp. This, in context with Question 9, which focused on the feel of exposing the pulp between the 3D-printed tooth and extracted teeth, had an overall score of 1.45 (±0.13), meaning students “strongly agree[d]” that the 3D-printed teeth were similar to that of extracted, yet the commercial teeth were not similar.

**Table 2:** Likert-scale responses of the students’ perceptions of the 3D-printed teeth with simulated canals.

Question no.	Fourth-year responses			Fifth-year responses			Overall
	$\bar{x}\pm \mathrm{SD}$	Min	Max	$\bar{x}\pm \mathrm{SD}$	Min	Max	$\bar{x}\pm \mathrm{SD}$
3.	1.27 ± 0.30	1	4	1.62 ± 0.32	1	4	1.46 ± 0.16
4.	2.03 ± 0.53	1	4	2.71 ± 0.43	2	5	2.39 ± 0.25
5.	2.08 ± 0.48	1	4	2.12 ± 0.46	1	4	2.10 ± 0.23
6.	2.95 ± 0.73	1	4	3.98 ± 0.82	1	5	3.50 ± 0.40
7.	1.30 ± 0.29	1	4	1.72 ± 0.21	1	4	1.53 ± 0.13
8.	3.89 ± 0.79	1	5	3.79 ± 0.63	2	5	3.84 ± 0.35
9.	1.27 ± 0.30	1	4	1.60 ± 0.22	1	4	1.45 ± 0.13
10.	4.38 ± 0.30	1	5	3.84 ± 0.77	1	5	4.09 ± 0.49
11.	1.42 ± 0.27	1	4	1.98 ± 0.34	1	4	1.72 ± 0.16
12.	1.51 ± 0.30	1	4	2.09 ± 0.46	1	4	1.83 ± 0.21
13.	1.78 ± 0.40	1	4	2.51 ± 0.39	1	5	2.18 ± 0.20

Numerical values were assigned to the responses; “strongly agree” (1), “agree” (2), “neither agree or disagree” (3), “disagree” (4) and “strongly disagree” (5).

In response to the 11 Likert-scale questions, overall, five questions had an average response corresponding to “strongly agree” (Questions 3, 7, 9, 11 and 12), and three questions corresponded to “agree” (Questions 4, 5 and 13), two questions corresponded to “neither agree or disagree” (Questions 6 and 8) and one question, Question 10, the response corresponded to “strongly disagree”.

**Figure 3:**
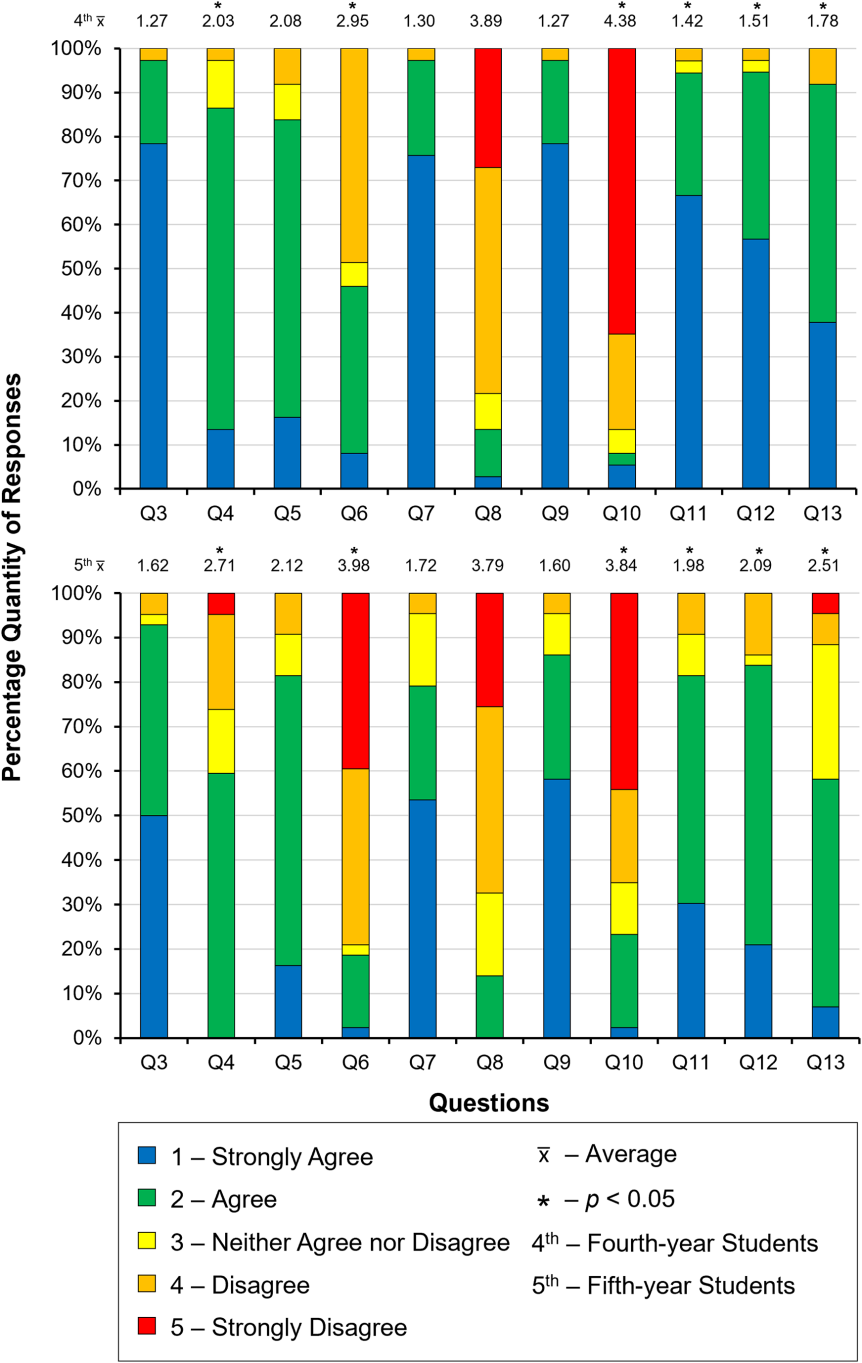
Distribution of the responses to the Likert-scale questions from both fourth- (top) and fifth- (bottom) year students.

Figure 3 shows the distribution of results collected showing the similarities and differences between the two year groups. Questions 3, 5, 7, 8 and 9 showed no statistical difference between the year groups, suggesting the responses were similar. However, Questions 4, 6, 10, 11, 12 and 13 were found to be all statistically significant (*p *< 0.05) between year groups, suggesting a difference in response between fourth- and fifth-year students. When comparing the 3D-printed typodont to either extracted teeth or the commercial typodont tooth, there was a significant difference between Questions 5 and 6, 7 and 8, and 9 and 10.

The biggest difference in responses between year groups was seen in Question 6, in relation to the likeness of drilling the enamel, when comparing the 3D-printed tooth with the commercial tooth. Statistical analysis showed a significant difference (*p *= 0.0034) between the 2 year- groups' responses, the fourth-year's response, on average, was 2.95 (±0.73) an “agree” rating, whereas the fifth-year's response was 3.98 (±0.82) with a “neither agree or disagree”. In context, Question 5, compared the enamel of the 3D-printed teeth with extracted, on average students “agree[d]” that the two were similar (2.10 ± 0.23). Overall, between extracted teeth and the 3D-printed typodont tooth, students “agree[d]” it was similar, but in comparison to the commercial typodont tooth, students “neither agree[d] or disagree[d]” that they were similar in likeness of drilling. Suggesting that the 3D-printed enamel more closely resembled that of the extracted, compared with the commercial.

Question 8 showed no significant difference (*p *= 0.056) statistically, and had the most similar response between year-groups, 3.89 (±0.79) and 3.79 (±0.63) for fourth- and fifth-years respectively. Question 8 was in relation to the likeness of drilling the dentine between the 3D-printed typodont with the commercial typodonts. However, when comparing the 3D-printed typodont tooth to either extracted or commercial typodont dentine, there was a significant difference (*p *= 0.0011), with students overall saying “agree[ing]” that the experience of drilling the dentine the 3D-printed typodont tooth is like extracted (Question 7), but “neither agree[d] or disagree[d]” it was similar to the commercial typodont tooth. Suggesting the 3D-printed typodont dentine was more similar to extracted than the commercial.

The largest difference in terms of “feel” was seen in Questions 9 and 10, about the likeness of exposing the pulp. Overall, students “strongly agree[d]” that exposing the pulp on the 3D-printed typodont tooth was similar to extracted teeth, but in comparison to the commercial typodont tooth, students “disagree[d]” that it was similar to the commercial typodont tooth, this difference in perception was significantly different (*p *= 0.0013).

Within the year-groups, both Questions 8 and 10, were significantly different (*p* = 0.0005 and *p* = 0.0027, respectively) in responses compared with the other question responses. The fifth year's response to Question 6, was also significantly different statistically (*p *= 0.0052), compared with the responses to the other questions.

**Figure 4:**
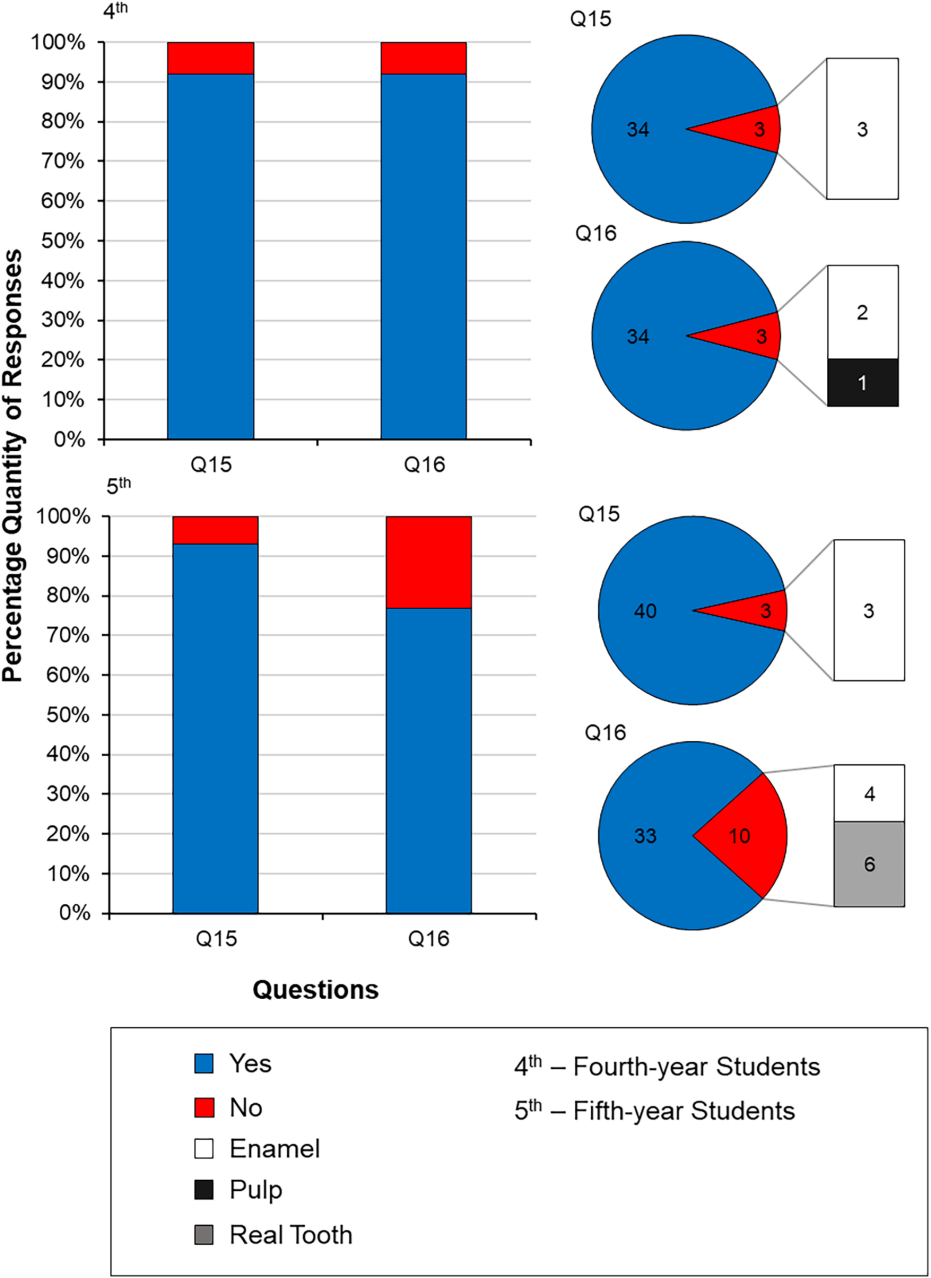
Distribution of the responses to questions 15 (would you use this tooth in endodontics CSL technique courses?) and 16 [would you prefer having this tooth in assessments (including gateway and revalidation tests)?] from both fourth- and fifth-year undergraduate dental students. If a “no” response was given in Questions 15 and 16, the participants justified their responses by stating their dissatisfaction with either the enamel material, the size of the pulp, or that they would prefer the use of a real tooth in Endodontics CSL Technique courses and Gateway and Revalidation tests.

Figure 4 shows the distribution of the results from questions 15 to 16. Overall, 92.5% of participants (92% fourth- and 93% fifth-years), said yes to using the 3D-printed typodonts in their Endodontics Clinical Skills Laboratory Technique course (Question 15), and 84.5% also said yes (92% fourth- and 77% fifth-years) to using the teeth in assessments (Question 16).

Regarding Question 15 (the use in the Clinical Skills Laboratory Technique course in Endodontics), there was no significant difference (*p *= 0.058) between the responses of the fourth- and fifth-year students. For both year groups, the same answer was given, if the participants answered “no” to the question, the reason being that the enamel was why they chose to answer “no”. Question 16 (use in assessments), however, showed a significant difference in the “yes” response between year groups, with 92% of fourth-year students saying “yes” to using the 3D-printed typodonts in their assessments, compared with only 77% of fifth-years. The reasoning behind the “no” responses, for the fourth-year students was similar to that of Question 15, and the enamel was given as to the reason why, along with the pulp also being a factor. The fifth-year students also stated the enamel being the reason for their negative responses, however, a larger majority stated that they would prefer to use real extracted teeth in their assessments compared to the 3D-printed ones.

Between both years, responses to Question 17 (the favourite aspect of the 3D-printed tooth), mostly focussed on the texture and feel of the overall tooth (35%), in particular cutting the dentine (19%), along with exposing the pulp (19%), listing these two factors as their most favoured aspect of the 3D-printed tooth (Table 3). The fact the anatomy was based on a real tooth, was also noted as being a popular answer given (19%). In terms of the responses to Question 18 (least favourite aspect), the most common response from participants from both year groups, was the feel and texture of enamel (38%), with most students commenting that the enamel felt “absent” when cutting. Another common response was the location of the pulp (38%), students noted that the location was different when compared to the commercial tooth that they use (Real T Endo, USA).

**Table 3:** Responses to the “free-text” comments, questions 17 (what aspect of the model was your most favourite?) and 18 (what aspect of the model was your least favourite?).

Question no.	Responses	Fourth-year response	Fifth-year response	Overall response
17.	“Realistic anatomy”	7 (21%)	7 (17%)	14 (19%)
	“The feel and texture of the enamel”	2 (6%)	4 (10%)	6 (8%)
	“The feel and texture of the dentine”	7 (21%)	7 (17%)	14 (19%)
	“The feel of exposing the pulp”	6 (18%)	8 (20%)	14 (19%)
	“Overall feel and texture of the tooth”	11 (33%)	15 (37%)	26 (35%)
18.	“Unrealistic anatomy”	5 (21%)	0 (0%)	5 (12%)
	“The feel and texture of the enamel”	6 (25%)	10 (56%)	16 (38%)
	“The feel and texture of the dentine”	1 (4%)	0 (0%)	1 (2%)
	“Location of the pulp”	8 (33%)	8 (44%)	16 (38%)
	“Overall feel and texture of the tooth”	4 (17%)	0 (0%)	4 (10%)

The number of responses for each year group given, as well as the percentage of that response.

Responses to Question 19 were low (8%), however, the responses reiterated students' answers in Questions 15 and 16, with those having favourable outcomes, they would like to use the 3D-printed typodonts again. Those that provided more comments, suggested that they would prefer to use the 3D-printed typodonts provided over the commercial teeth throughout the undergraduate degree.

## Discussion

This study was the first to evaluate the Years 4 and 5 undergraduate dental students' perceptions of the developed 3D-printed teeth. The central incisor teeth with simulated root canal systems (Real T Endo, Acadental, Inc., USA) were chosen as a comparison due to their widespread familiarity and regular use within the dental school's curriculum. The Acadental teeth acted as a reference point that students were accustomed to working with during their training. Allowing a direct comparison of the perceptions of students when exposed to a familiar model vs. a novel 3D-printed alternative. This comparative approach allowed for the assessment of the acceptability and potential advantages of 3D-printed typodonts within the context of existing dental education practices. However, is it worth acknowledging the potential bias introduced by students’ familiarity with Acadental teeth. Future studies may benefit from testing the 3D-printed typodonts against another commercially available tooth with similar mechanical properties to obtain more unbiased feedback. The study would have also benefited from evaluating other haptically similar typodont teeth, however, there is limited literature on the production and viability of these typodont teeth.

Out of a total of 143, 37 fourth-year and 43 fifth-year dental students, 80 questionnaires were returned, resulting in a response rate of 55.94%. Each participant was requested to evaluate their training experiences with the 3D-printed typodonts. The response rate was deemed acceptable for this study, considering the voluntary nature of participation and the relatively large sample size obtained. However future studies would look to increase the sample size for larger data collection.

This study demonstrated that on average fifth-year students would tend to give a lower overall rating, compared to fourth-year students. This was seen in Questions 4, 6, 10–12 and 13 when the responses were significantly different between the 2 year groups. This could be a factor of experience, in the sense, that fifth-year students have had 3 years of experience when dealing with endodontic techniques in comparison to the fourth-year's 2 years of experience. This greater experience means that fifth-year students have been exposed to using extracted teeth more often, demonstrating a wider knowledge of what cutting them “feels” like, therefore, having an affinity to using them in their education. This was evident in the responses to Question 16, with students answering “no” and stating the reason for using real teeth in their Gateway and Revalidation assessments. It should be noted that all students are required to complete Clinical Skills Laboratory technique courses in Endodontics before being allowed to undertake Gateway assessments to ensure that dental students demonstrate safe practice before being permitted to treat patients in the clinic. Student attainment is also reviewed yearly with revalidation assessments to maintain their competency in clinical skills by being assessed and then provided with structured feedback on phantom heads at the Clinical Skills Laboratory before patient treatment.

The study aimed to assess the differences in tactile perception among natural, commercially available, and 3D-printed typodont teeth. Distinct characteristics in the tactile feedback were experienced when interacting with each type of tooth. The students stated that the 3D-printed tooth was easier to cut, and when likened to extracted teeth, the 3D-printed tooth ranked high in comparison to the Real T Endo tooth. This vast difference in tactile similarity is most likely due to the amount of force needed to cut the tooth, as previously established (13). With extracted enamel requiring 0.31 N (±0.12) and the 3D-printed enamel requiring 0.36 N (±0.03), whereas commercially available typodont enamel ranged from 0.69 to 1.13 N. This difference in force is reflected in the students' perception of ease of cutting as well as the tactile similarity between the extracted and 3D-printed teeth.

In Table 2, responses were assigned to numerical values; “strongly agree” (1), “agree” (2), “neither agree or disagree” (3), “disagree” (4) and “strongly disagree” (5). The response to the likeness of drilling the enamel (Question 5), in terms of feel, received a similar response from both fourth- (2.08 ± 0.48) and fifth-year (2.71 ± 0.43) students, with participants “agree[ing]” that the feel was similar to that of extracted enamel (2.10 ± 0.23). When comparing the 3D-printed typodont with the commercial tooth, the fourth-year participants “agree[d]” that the likeness was similar (2.95 ± 0.73), whereas, the fifth-year students “neither agree[d] or disagree[d]” that the likeness was similar (3.98 ± 0.82). In terms of tactile response, the 3D-printed typodonts were ranked similarly to extracted enamel, compared to commercially available enamel. This corresponds with what was found in Cresswell-Boyes et al. (13).

Questions 7 and 8 compared the likeness of drilling the dentine of the 3D-printed typodont with extracted and commercial teeth. The responses for both the fourth- and fifth-year students were similar to each question, especially for students “strongly agree[ing]” that the 3D-printed dentine was like that of extracted dentine (1.53 ± 0.13). Whereas, when compared with the commercial teeth, the participants “neither agree[d] or disagree[d]” that the two teeth were alike (3.84 ± 0.35). Tactile perception from these questions corroborates what was established previously (13), with the participants agreeing that the 3D-printed tooth is similar to cutting extracted teeth in comparison to the commercial ones, suggesting the commercial typodont teeth are not haptically-similar to natural teeth.

Participants rated the “feel” of exposing the pulp chamber in the 3D-printed teeth highly (Question 9), with both fourth- and fifth-year students “strongly agree[ing]” that this was similar to that of extracted teeth (1.45 ± 0.13). When compared to the commercial teeth (Question 10), fourth-year students “disagree[d]” that the teeth were alike (4.38 ± 0.30), and the fifth-years “neither agree[ing] or disagree[ing]” that the teeth were alike (3.84 ± 0.77). The responses between the extracted and commercial teeth demonstrated a statistical difference (*p = *0.0027) since the participants agreed that the 3D-printed typodonts closely matched that of extracted teeth when exposing the dental pulp; highlighting a tactile similarity between the 3D-printed typodonts and natural teeth.

Overall, 92.5% of students indicated that they would be happy to use the 3D-printed typodonts again in future laboratory-based techniques courses and assessments (Question 15), and 84.5% reported that they would be happy to use them in their gateway and revalidation exams before patient treatments [Question 16 (Figure 4)], suggesting that the 3D-printed typodont is a popular choice amongst students. However, 7.5% stated “no” to Question 15, and 15.5% to Question 16, with both answers stating the enamel was the reason for not choosing the 3D-printed one; in Question 16, 78% of the “no” responses stated this reason for their preference to use extracted teeth compared to any artificial teeth.

In addition, it was noted that students that spent less time (<5 min) in preparing the access cavities (Question 14) had given “no” responses in either Questions 15 or 16, whereas, students that spent longer (>5 min) were more likely to answer “yes” to these questions. It can be speculated that students failed to take their time to evaluate these teeth. Interestingly, this was also seen to be the case with Questions 3–13, in those students that took less time evaluating the 3D-printed teeth, would rate the tooth lower against extracted and commercial teeth.

One of the limitations of this study is the lack of focus groups to receive more in-depth feedback and understand how the students perceived the 3D-printed teeth in comparison to the traditional artificial ones. In future, it would also be beneficial to include Year 3 dental students, as this is typically when students are introduced to the Basic Endodontics Technique course. This would provide beginners' feedback to validate the current findings by comparing their responses with the experienced (fourth and fifth-year) students'. Although this study assessed students' perceptions of the tactile characteristics of natural, commercially available, and 3D-printed typodont teeth, it did not include a quantitative assessment of the endodontic procedures performed. While subjective feedback offers valuable insights, future research should consider incorporating objective measures to evaluate procedural efficacy and precision.

Previous studies have explored various 3D printing materials for dental applications, including composite resins and commercially available products such as Rigid Resin and White Resin (Formlabs Inc., United States). These materials have shown promise in creating realistic carious lesions and dental pulp cavities (18) and modular training models (19). However, despite these advancements, there remains no commercially available material specifically designed for printing teeth that accurately mimics the mechanical properties and tactile sensation of natural dentition. This study aimed to address this gap by offering an alternative material specifically engineered for recreating teeth, ensuring a more realistic and effective training tool for dental education.

## Conclusion

Within the limitations of this study, the 3D-printed typodonts were rated highly in comparison to the commercial teeth (Real T Endo) about overall operative experiences, suggesting that the participants would prefer to use the printed typodonts. However, it should be noted that the enamel material still needs improvements in terms of tactile sensation and aesthetics. The ease at which these 3D-printed teeth were produced, demonstrates the potential for these to be produced “in-house” within dental institutes and offers an alternative material to those that have been previously published. The future work will focus on making the micro-CT models and methodologies freely available for dental schools, as well as, focussing on improving the enamel material by utilising different materials or post-processing techniques.

## Data Availability

The original contributions presented in the study are included in the article/Supplementary Material, further inquiries can be directed to the corresponding author.

## References

[1] De Moor J Hülsmann M Kirkevang L-L Tanalp J Whitworth J. Undergraduate curriculum guidelines for endodontology. Int Endod J. (2013) 46(12):1105–14. 10.1111/iej.12186 24117830

[2] Leoni GB Versiani MA Pécora JD de Sousa-Neto MD. Micro-computed tomographic analysis of the root canal morphology of mandibular incisors. J Endod. (2014) 40:710–7. 10.1016/j.joen.2013.09.003 24767569

[3] Pouhaër M Picart G Baya D Michelutti P Dautel A Pérard M Design of 3D printed macro-models for undergraduates’ preclinical practice of endodontic access cavities. Eur J Dent Educ. (2021) 26:347–53. 10.1111/eje.12709 34358393

[4] Qualtrough AJ Whitworth JM Dummer PM. Preclinical endodontology: an international comparison. Int Endod J. (1999) 32(1):406–14. 10.1046/j.1365-2591.1999.00253.x 10551115

[5] Sennhenn-Kirchner S Goerlich Y Kirchner B Notbohm M Schiekirka S Simmenroth A The effect of repeated testing vs repeated practice on skills learning in undergraduate dental education. Eur J Dent Educ. (2018) 22(1):e42–7. 10.1111/eje.12254 28117541

[6] Nassri MR Carlik J da Silva CR Okagawa RE Lin S. Critical analysis of artificial teeth for endodontic teaching. J Appl Oral Sci. (2008) 16(1):43–9. 10.1590/S1678-77572008000100009 19089288 PMC4327279

[7] Tchorz JP Brandl M Ganter PA Karygianni L Polydorou O Vach K Pre-clinical endodontic training with artificial instead of extracted human teeth?: does the type of exercise have an influence on clinical endodontic outcomes? Int Endod J. (2015) 48:888–93. 10.1111/iej.12385 25266846

[8] Bitter K Gruner D Wolf O Schwendicke F. Artificial versus natural teeth for preclinical endodontic training: a randomized controlled trial. J Endod. (2016) 42(8):1212–7. 10.1016/j.joen.2016.05.020 27469437

[9] Costamagna P Carpegna G Bianchi C Baldi A Pasqualini D Scotti N Endodontic treatment of a molar with peculiar anatomy: case study with CBCT and 3D printed model. J Contemp Dent Pract. (2022) 22:1477–82. 10.5005/jp-journals-10024-3175 35656690

[10] Smutný M Kopeček M Bezrouk A. An investigation of the accuracy and reproducibility of 3D printed transparent endodontic blocks. Acta medica (Hradec Kralove). (2022) 65:59–65. 10.14712/18059694.2022.19 36458933

[11] Zhang R Tang R Spintzyk S Tian Y Xiang Y Xu Y Three-dimensional printed tooth model with root canal ledge: a novel educational tool for endodontic training. Dent J. (2023) 11:213. 10.3390/dj11090213 PMC10527572 37754333

[12] Al-Sudani DI Basudan SO. Students’ perceptions of pre-clinical endodontic training with artificial teeth compared to extracted human teeth. Eur J Dent Educ. (2016) 21(4):e72–5. 10.1111/eje.12223 27495270

[13] Cresswell-Boyes AJ Davis GR Krishnamoorthy M Mills D Barber AH. Composite 3D printing of biomimetic human teeth. Sci Rep. (2022) 12(1):7830. 10.1038/s41598-022-11658-y 35550557 PMC9098645

[14] Reymus M Stawarczyk B Winkler A Ludwig J Kess S Krastl G A critical evaluation of the material properties and clinical suitability of in-house printed and commercial tooth replicas for endodontic training. Int Endod J. (2020) 53(10):1446–54. 10.1111/iej.13361 32623735

[15] Garcia-Sanchez A Mainkar A Bakhsh K Sanchez S Tadinada A Chen IP. The use of three-dimensional (3D)-printed guide for identifying root canals in endodontic treatment. Sci Inquest. (2020) 3(1):104.

[16] Reis T Barbosa C Franco M Batista C Alves N Castelo P 3D-printed teeth in endodontics: why, how, problems and future-a narrative review. Int J Environ Res Public Health. (2022) 19:7966. 10.3390/ijerph19137966 35805624 PMC9265401

[17] Oza S Lai G Peters O Chen J Karabucak B Scott R The influence of CBCT-derived 3D-printed models on endodontic microsurgical treatment planning and confidence of the operator. J Endod. (2023) 49:521–7.e2. 10.1016/j.joen.2023.02.004 36804199

[18] Höhne C Schwarzbauer R Schmitter M. 3D printed teeth with enamel and dentin layer for educating dental students in crown preparation. J Dent Educ. (2019):1446–54. 10.1111/iej.13361 83(12):1457–63. doi: 10.21815/JDE.019.146 83(12):1457–63. doi: 10.21815/JDE.019.146 31451556

[19] Hanafi A Donnermeyer D Schäfer E Bürklein S. Perception of a modular 3D print model in undergraduate endodontic education. Int Endod J. (2020) 53(7):1007–16. 10.1111/iej.13299 32220071

[20] Tonini R Xhajanka E Giovarruscio M Foschi F Boschi G Atav-Ates A Print and try technique: 3D-printing of teeth with Complex anatomy a novel endodontic approach. Appl Sci. (2021) 11(4):1511. 10.3390/app11041511

[21] Kolling M Backhaus J Hofmann N Keß S Krastl G Soliman S Students’ perception of three-dimensionally printed teeth in endodontic training. Eur J Dent Educ. (2021) 00:1–9. 10.1111/eje.12743 34921718

[22] Vertucci FJ. Root canal anatomy of the human permanent teeth. Oral Surg Oral Med Oral Pathol Oral Radiol. (1984) 58(8):589–99. 10.1016/0030-4220(84)90085-9 6595621

[23] Cresswell-Boyes AJ Barber AH Mills D Tatla A Davis GR. Approaches to 3D printing teeth from x-ray microtomography. J Microsc. (2018) 272(3):207–12. 10.1111/jmi.12725 29953620

[24] Cohen S Hargreaves KM. Pathways of the Pulp. St. Louis, Missouri: Mosby Elsevier (2006).

[25] Landi E Gelotti G Logroscino G Tampieri A. Carbonated hydroxyapatite as bone substitute. J Eur Ceram Soc. (2003) 23(15):2931–7. 10.1016/S0955-2219(03)00304-2

