# Corrigendum: Students’ perceptions of endodontic typodont teeth with simulated canals printed from novel materials

**DOI:** 10.3389/fdmed.2024.1547849

**Published:** 2025-01-07

**Authors:** Alexander Jon Cresswell-Boyes, Graham Roy Davis, Aylin Baysan

**Affiliations:** ^1^ Peninsula Dental School, Faculty of Health, University of Plymouth, Plymouth, United Kingdom; ^2^ Dental Physical Sciences Unit, Centre for Oral Bioengineering, Institute of Dentistry, Barts and the London School of Medicine and Dentistry, Queen Mary University of London, London, United Kingdom; ^3^ Centre for Oral Bioengineering, Institute of Dentistry, Barts and the London School of Medicine and Dentistry, Queen Mary University of London, London, United Kingdom

**Keywords:** dental education, 3D printing, endodontics, simulation-based education, haptic (tactile) perception

**A Corrigendum on**
Students’ perceptions of endodontic typodont teeth with simulated canals printed from novel materials By Cresswell-Boyes AJ, Davis GR and Baysan A. (2024). Front. Dent. Med. 5:1373922. doi: 10.3389/fdmed.2024.1373922

**Text Correction**

In the published article, an error occurred in the order of the enamel and dentine descriptors.

A correction has been made to **Materials & methods**, *Fabrication of the 3D-printed teeth*, Paragraph 4. This sentence previously stated:

“An Anycubic Photon 3D printer [Anycubic, China (Figure 2C)] and it's proprietary slicing software [Anycubic Photon Slicer, Version 1.3.3, 2018, Anycubic, China (Figure 2B)] was then utilised in the production of the 3D-printed typodonts with the enamel and dentine composed of carbonated hydroxyapatite resins (5 wt.% and 20 wt.% respectively) with Anycubic White Resin (Anycubic, China)”

The corrected sentence appears below:

“An Anycubic Photon 3D printer [Anycubic, China (Figure 2C)] and it's proprietary slicing software [Anycubic Photon Slicer, Version 1.3.3, 2018, Anycubic, China (Figure 2B)] was then utilised in the production of the 3D-printed typodonts with the dentine and enamel composed of carbonated hydroxyapatite resins (5 wt.% and 20 wt.% respectively) with Anycubic White Resin (Anycubic, China)”

The authors apologize for this error and state that this does not change the scientific conclusions of the article in any way. The original article has been updated.

